# Differentiation between infectious spondylodiscitis versus inflammatory or degenerative spinal changes: How can magnetic resonance imaging help the clinician?

**DOI:** 10.1007/s11547-021-01347-7

**Published:** 2021-04-02

**Authors:** Fausto Salaffi, Luca Ceccarelli, Marina Carotti, Marco Di Carlo, Gabriele Polonara, Giancarlo Facchini, Rita Golfieri, Andrea Giovagnoni

**Affiliations:** 1 Clinica Reumatologica, Ospedale “Carlo Urbani”, Dipartimento Di Scienze Cliniche E Molecolari, Università Politecnica Delle Marche, Via Aldo Moro, 25, 60035 Jesi, Ancona Italy; 2 Unità di Radiologia, Dipartimento di Medicina Specialistica, Diagnostica E Sperimentale, Ospedale Sant’Orsola, Università Di Bologna, Via Albertoni 15, 40138 Bologna, Italy; 3 Dipartimento di Scienze Radiologiche, Ospedali Riuniti, Università Politecnica Delle Marche, Ancona, Italia; 4 Radiologia Diagnostica ed Interventistica, IRCCS Istituto Ortopedico Rizzoli, Via GC Pupilli 1, 40136 Bologna, Italia

**Keywords:** Spondylodiscitis, Magnetic resonance imaging, Vertebral infection, Spondyloarthritides

## Abstract

Spondylodiscitis is a complex disease whose diagnosis and management are still challenging. The differentiation between infectious and non-infectious aetiology is mandatory to avoid delays in the treatment of life-threatening infectious conditions. Imaging methods, in particular magnetic resonance imaging (MRI), play a key role in differential diagnosis. MRI provides detailed anatomical information, especially regarding the epidural space and spinal cord, and may allow differential diagnosis by assessing the characteristics of certain infectious and inflammatory/degenerative lesions. In this article, we provide an overview of the radiological characteristics and differentiating features of non-infectious inflammatory spinal disorders and infectious spondylodiscitis, focussing on MRI results and presenting relevant clinical and pathological features that help early diagnosis.

## Introduction

Spondylodiscitis can be divided into two major macrocategories, namely non-infectious inflammatory spinal disorders and infectious diseases [1]. The definition of spondylodiscitis in the strict sense applies to infectious diseases; however, there are several non-infectious conditions that can mimic the presence of an infectious vertebral disease. The distinction between inflammatory/degenerative versus infectious pathology has a huge prognostic impact. The correct diagnosis of infection basically requires two main criteria: the presence of characteristic lesions in the spine and the isolation of the pathogen from blood or infected site. Therefore, radiological evaluation is important both for the diagnosis of spondylodiscitis and for further planning and monitoring of treatment.

Magnetic resonance imaging (MRI) is considered the imaging modality of choice for the detection and evaluation of spondylodiscitis, with a sensitivity of 96% and specificity of 92% in the diagnosis of infectious processes [2].

In this article, an overview of radiological appearances of non-infectious inflammatory spinal column disorders and infectious spondylodiscitis has been provided, focussing on MRI results and presenting relevant clinical and pathological features that help early and differential diagnosis.

## Inflammatory disorders of the spine

A variety of inflammatory non-infectious disorders may involve the spine. Of these, axial spondyloarthritis (axSpA), synovitis–acne–pustulosis–hyperostosis–osteitis (SAPHO) syndrome, active discopathy-related spinal abnormalities (named Modic type 1 lesion), calcific disease or spinal gout are the most commonly encountered causes of spondylodiscitis. Destructive spondyloarthropathies can also be seen in patients with a story of long-term haemodialysis (Table 1).

**Table 1:** Inflammatory non-infectious disorders of the spine and infectious aetiology of pyogenic spondylodiscitis

Inflammatory non-infectious disorders of the spine	Infectious spondylodiscitis
Spondyloarthritides [axSpA]*	Pyogenic vertebral osteomyelitis Gram-positive aerobic cocci Staphylococcus aureus, Staphylococcus epidermidis, Haemophilus influenzae, Streptococcus pyogenes, Enterococcus spp., other streptococci Gram-negative aerobic bacilli Escherichia coli, Proteus spp., Pseudomonas aeruginosa, Klebsiella pneumoniae, Enterobacter spp., Salmonella
Synovitis–acne–pustulosis–hyperostosis–osteitis [SAPHO]	
Modic changes type-1 syndrome	
Acute symptomatic calcific discitis Spinal gout Destructive spondyloarthropathy of haemodialysis	
	Infectious granulomatous diseases Tuberculous spondylodiscitis Brucella spondylodiscitis
	Fungal infection Candida spp., Aspergillus spp., Cryptococcus, Coccidioides immitis, Blastomyces dermatitidis
	Parasitic spinal infections Taenia solium, Schistosoma japonicum, S. mansoni, S. haematobium, Toxoplasma gondii, Echinococcus granulosus

*axSpA comprise ankylosing spondylitis (AS), reactive arthritis, arthritis/spondylitis in inflammatory bowel diseases (IBD), and psoriatic arthritis with axial involvement

### Axial spondyloarthritis

**Fig. 1:**
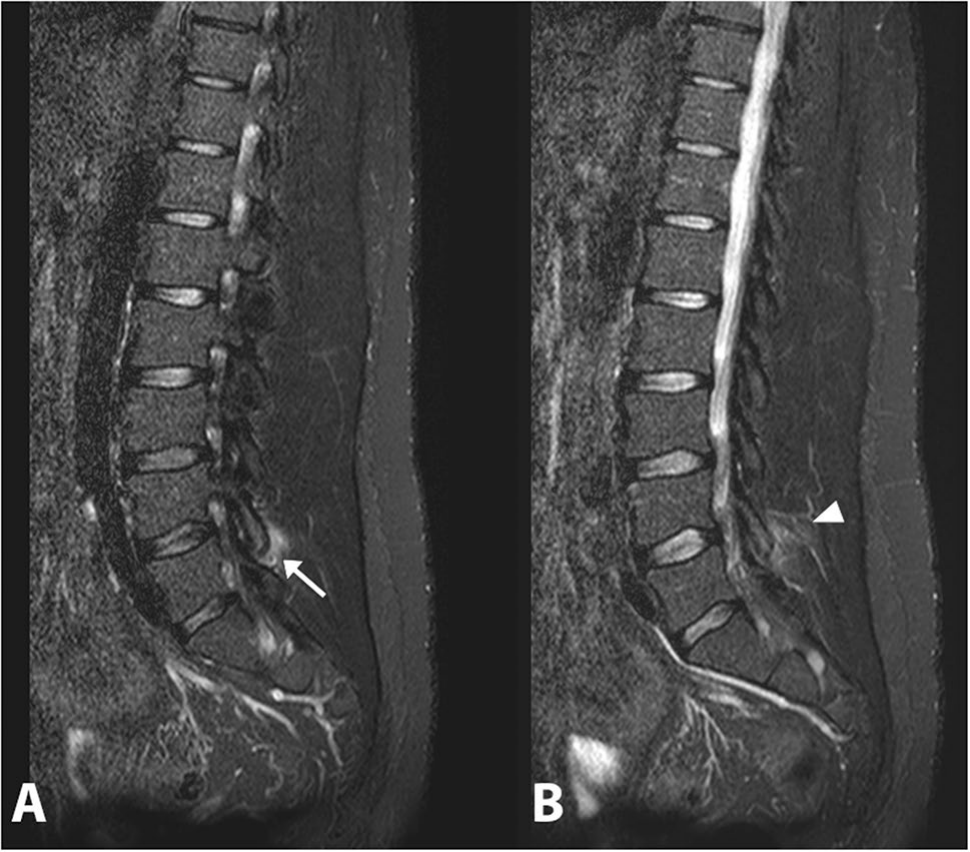
Sagittal STIR images (**a, b**) active inflammation of the left facet joint at L4-L5 (arrow). Inflammation of the adjacent soft tissue can also be observed (arrowhead)

Ankylosing spondylitis (AS), psoriatic arthritis, reactive arthritis and enteropathic arthritis are included in the context of axSpA [3]. MRI has radically changed the diagnostic approach of these conditions, to the extent that the latest classification criteria for axSpA, developed by the Assessment of SpondyloArthritis International Society (ASAS), have included MRI of the sacroiliac joints and spine for diagnostic/classification purposes [4]. The recommended approach for spine imaging in patients with axSpA includes T1-weighted sequences to assess the morphology of structures, T2-weighted sequences or short tau inversion recovery (STIR) to detect bone marrow edema (BME), and T1-weighted sequences suppressed with gadolinium to show tissue inflammation (enthesitis and synovitis) [5]. Characteristically, inflammatory lesions of the vertebral spine are present in several areas. Typically, inflammatory lesions of axSpA are found at the angular level (the presence of BME of the anterior vertebral angles is called Romanus lesions), at the central level, in the lateral and posterior spinal segments such as pedicles, costotransverse, costovertebral, and zigo-apophyseal joints (Fig. 1) [6]. The lesions may then tend towards erosive evolution in the anterior part of the thoracolumbar vertebral bodies [7] and, subsequently, erosions are associated with sclerotic changes and syndesmophytes which, in long-term disease, tend to fuse. Both active and structural lesions of the spine may be present in a patient simultaneously.

**Fig. 2:**
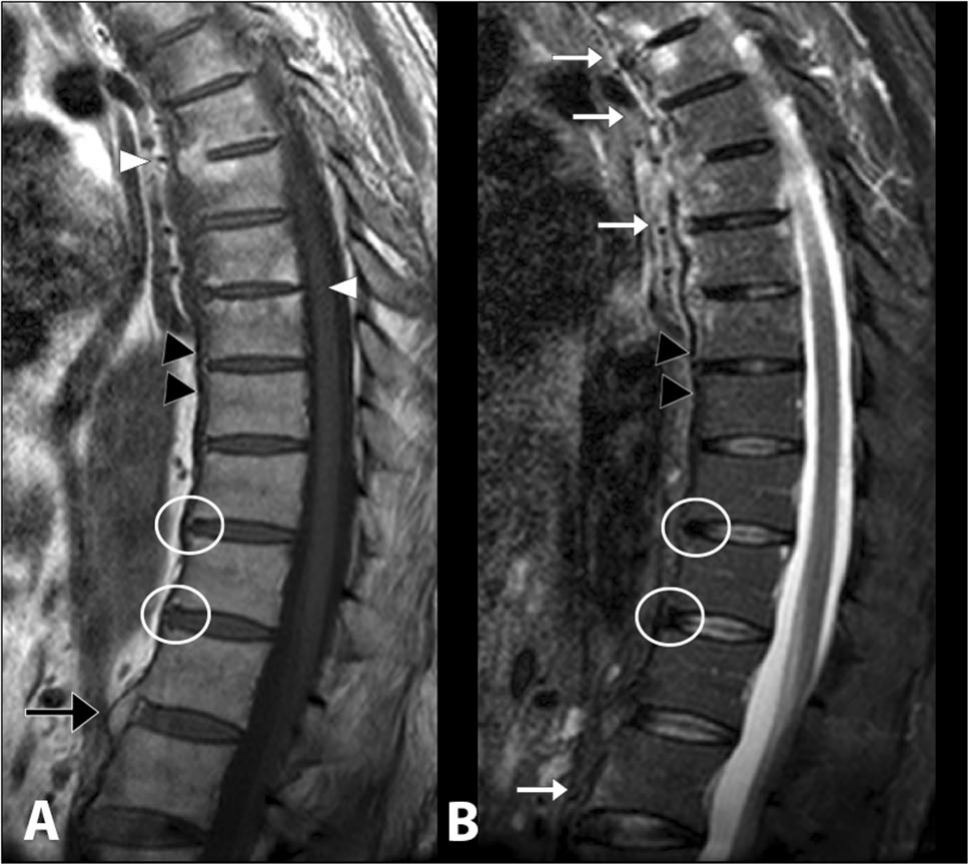
Sagittal T1-weighted image (**a**) and sagittal STIR image (**b**) showing hypointense and hyperintense lesions, respectively, at the anterior edges of vertebral bodies from D3 to D8 and in L1, indicating bone marrow edema (Romanus lesions) (white arrows). The post-inflammatory areas of fatty degeneration of the bone marrow are visible at the anterior edges of the opposite vertebral bodies D5/D6 and at the posterior edges of D7 and D8 (white arrowheads). Thickening of the anterior longitudinal ligament (black arrowheads) and multiple syndesmophytes (white circles) can also be detected. Furthermore, in D12-L1 it is possible to observe a bony bridge (black arrow), which could result from the progression of ossification on syndesmophytes

MRI is the only imaging tool capable of visualizing BME [8]. At these anatomical points, BME indicates active inflammation and consequently is highly suggestive of axSpA presence. However, specificity of BME in the diagnosis of axSpA is limited, since BME can be expression of mechanical back pain even in young patients [9]. Changes such as fat infiltration, erosion, or ankylosis indicate structural damage [10]. Romanus lesions are more clearly identified in the sagittal plane and are characterized by the presence of triangular-shaped BME at the corners of the vertebral endplates, with low signal on T1-weighted images and high signal on STIR and T2-fat suppressed sequences (Fig. 2). A spinal 'positive MRI' is defined by Outcome Measures in Rheumatology Clinical Trials (OMERACT) and the ASAS group when at least three inflammatory lesions of the anterior and/or posterior corners (anterior or posterior spondylitis) or fat deposits at the vertebral corners are present [11].

Occasionally, axSpA are accompanied by erosive focal changes in the vertebral terminal plate, defined as Andersson's lesions. These alterations are difficult to differentiate from the typical results of infectious spondylodiscitis. The incidence of Andersson's lesions is about 8–16%, commonly occurring in thoracolumbar segments [12]. MRI is fundamental in the differentiation between Andersson's lesions and infectious spondylodiscitis.

**Fig. 3:**
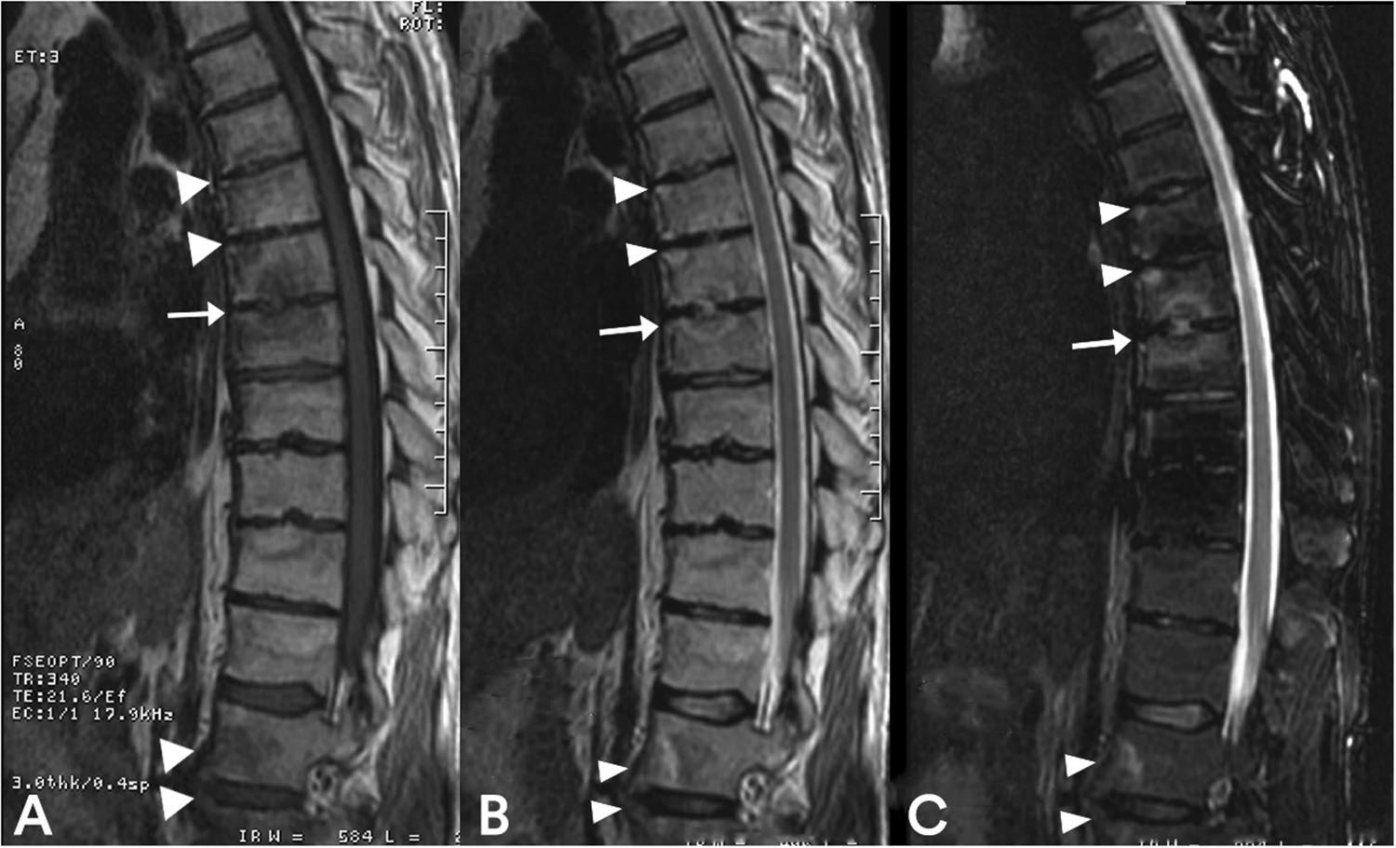
Sagittal T1-weighted image (**a**), sagittal T2-weighted image (**b**) and sagittal STIR image (**c**) showing a typical active Andersson lesion at D7/D8, with subchondral edema-osteitis, discitis and erosions (arrow). Romanus lesions can be observed in the anterior corners of the vertebral bodies at D6, D7 and at L1 and L2 (arrowheads)

In MRI, during the progression of bacterial spondylodiscitis, the disc often becomes a focal point for fluid collection. In axSpA, however, the disc usually retains its regular signal intensity or shows only signs of degeneration. Therefore, in axSpA, variations in signal intensity are likely to be limited within the vertebral body and the vertebral end plate, but not in the disc. Furthermore, perivertebral effusion and intradiscal effusions are rarely seen in discovertebral lesions in axSpA. The high signal intensity on T2-weighted images observed in Andersson's lesions usually corresponds to granulation tissue, and high-intensity T2 peripheral areas reflect infiltration of tissue and inflammatory cells, not fluid collection (Fig. 3). The lack of intradiscal or perivertebral fluid collection is an important diagnostic sign [13].

### Non-bacterial osteitis syndromes

Synovitis, acne, pustulosis, hyperostosis, and osteitis (SAPHO) syndrome and chronic recurrent multifocal osteomyelitis (CRMO) represent the spectrum of autoinflammatory bone diseases in which non-infectious osteitis is the unifying feature collectively termed non-bacterial osteitis syndrome (NOS). There is a striking overlap of symptoms and comorbidities between CRMO and SAPHO. CRMO may represent the paediatric presentation of SAPHO.

Under the term SAPHO syndrome are included chronic inflammatory disorders that have in common musculoskeletal manifestations characterized by the presence of synovitis, hyperostosis and osteitis, associated with characteristic skin manifestations such as neutrophilic rashes, palmo-plantar pustulosis or acne conglobata. The SAPHO syndrome can affect all ages. The most commonly affected skeletal region of the SAPHO syndrome is the anterior chest wall (ACW), including the sternoclavicular, manubriosternal and costosternal joints. The spine is the second most common site of skeletal involvement, and spinal abnormalities can be documented in up to 50% of adult patients [14].

Axial inflammatory manifestations can involve multiple sites and combine in different ways [15]. Elementary radiological lesions include angular lesions of the vertebral body, non-specific spondylodiscitis (very similar to infectious forms), osteolytic lesions with varying degrees of collapse of the vertebral body (visible lesions even in childhood), osteosclerosis of one or more vertebral bodies with development of hyperostosis, paravertebral ossification, and sacroiliitis (more common in adulthood) [15]. Angular erosions seen on MRI may suggest enthesitis and are somehow the equivalent of Romanus' lesion in axSpA. Similar to axSpA, active MRI lesions appear as BME and soft tissues edema [16]. With the attenuation of the inflammatory process, in MRI in T1-weighted sequences the lesions may become hyperintense rather than hypointense due to post-inflammatory fatty bone marrow degeneration [17]. Non-specific spondylodiscitis lesions of two or more contiguous vertebrae, with cortical erosions and underlying subchondral sclerosis in the endplates, on either side of an intervertebral disc, mimicking infectious spondylodiscitis, are usually located in the central or anterior part of the discovertebral junction [18]. The intervertebral disc space is usually well preserved; the height may be reduced. The low signal intensity of the disc on fluid-sensitive images and the absence of post-contrastographic enhancement of the disc space help to differentiate the SAPHO from infectious forms. However, the presence of both high signal intensity on T2-weighted images and post-contrastographic disc space enhancement is visible in up to 30% of cases [17]. The differential diagnosis of SAPHO syndrome compared to infectious spondylodiscitis may also be made difficult by the possible coexistence of prevertebral soft tissue swelling. SAPHO can be a multifocal disease, and total body MRI, with T1-weighted coronal sequences and STIR, is increasingly used to assess this condition [19].

CRMO consists of an autoinflammatory bone disorder that results in bone lesions, usually seen in children (approximately 7–12 years old, 2:1 female-to-male ratio) [20]. Patients usually have non-specific clinical findings and present with localized bone pain in the lower extremities, clavicle, and/or pelvis. One-third of patients has low grade fever. There is an association with other autoimmune disorders like inflammatory bowel diseases, psoriasis, and palmar plantar pustulosis [20].

CRMO is characterized by lytic lesion with a well-defined sclerotic hem. Bone lesions can occur anywhere throughout the body. However, they tend to have a predisposition to the metaphyseal region of bones (75%). Spinal involvement is not so rare and often involves different soma with bone edema and vertebral collapse [21]. On MRI, abnormal lesions have increased signal on the STIR sequence and decreased signal on the T1-weighted sequence. Whole body MRI is recommended to determine clinically silent lesions [22].

### Modic type 1 lesion

**Fig. 4:**
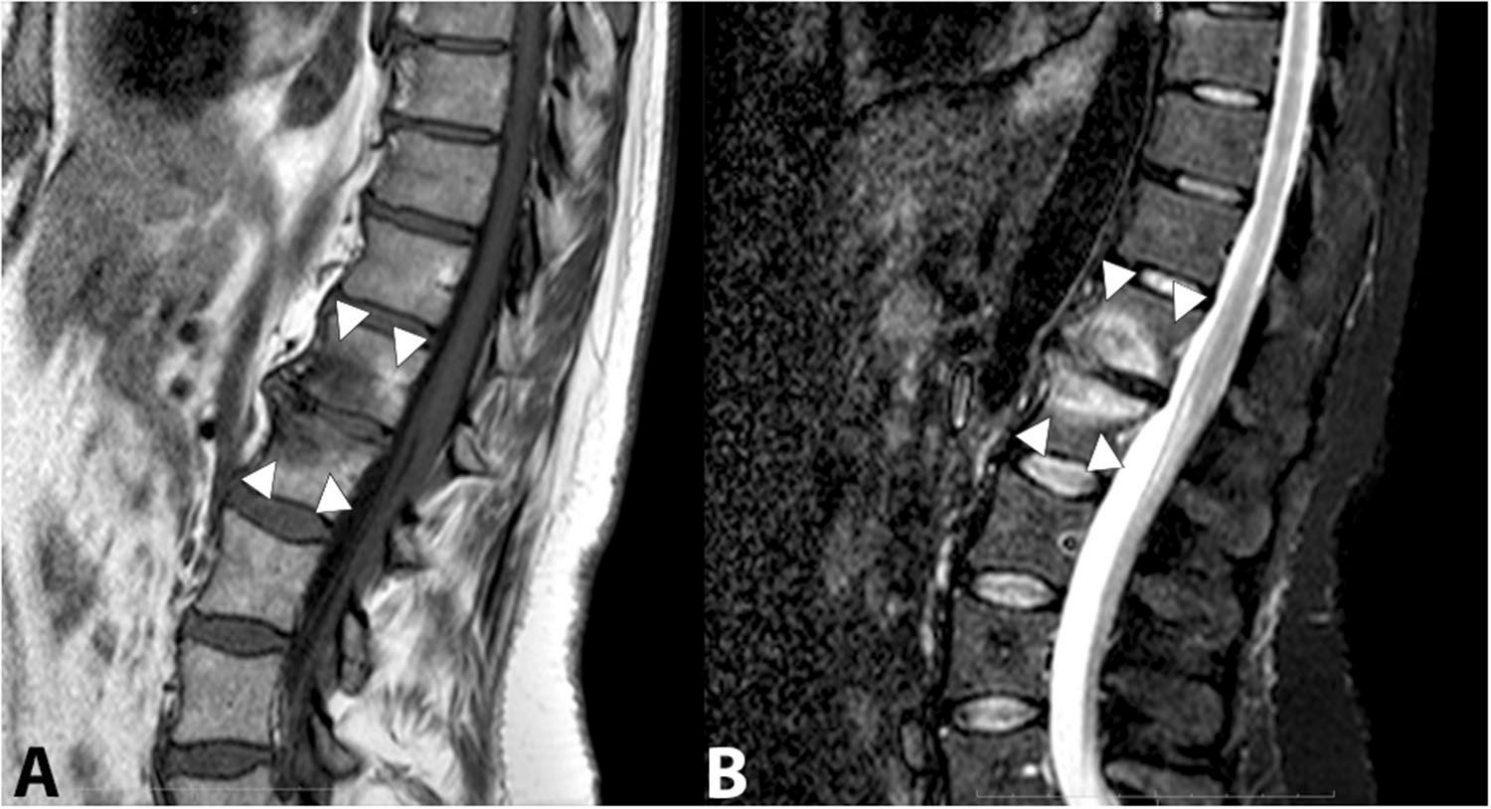
Sagittal T1-weighted image (**a**) and sagittal STIR image (**b**) showing fibrovascular and edematous pattern in the subchondral bone marrow of adjacent vertebral bodies L1/L2 (Modic type 1) (arrowheads). Bony degenerative changes can also be observed at the same level

Modic 1 lesions are alterations involving the subchondral bone of two adjacent vertebrae associated with degenerative disease of the intervertebral disc [23]. Modic 1 lesions are acute conditions, characterized in MRI by a hyperintensity of signal on T2- or STIR-weighted sequences and by hypointensity in T1-weighted sequences, compared to the bone marrow signal (Fig. 4).

**Fig. 5:**
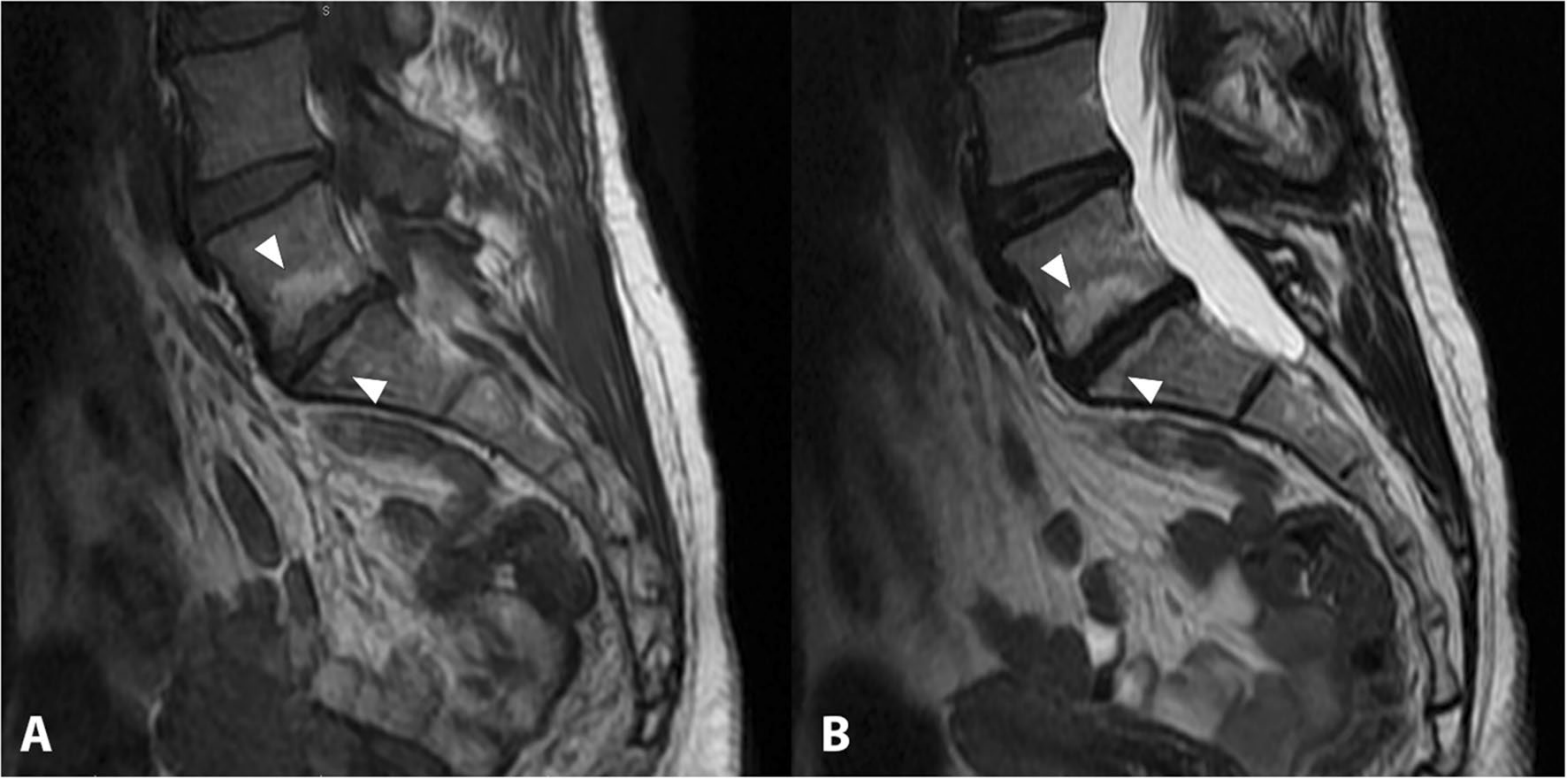
Sagittal T1-weighted image (**a**) and sagittal T2-weighted image (**b**) showing post-inflammatory area of fatty degeneration of the subchondral bone marrow of adjacent vertebral bodies L5/S1 (Modic type 2) (arrowheads)

Modic 2 lesions represent a chronic phase of the degenerative process, characterized by an isointense signal in T2- or STIR-weighted sequences and a higher signal intensity on T1-weighted sequences, considering the signal intensity always in relation to the bone marrow signal (Fig. 5).

Modic 3 lesions are the final stage of the degenerative process, and the main characteristic is the decrease in signal intensity in both STIR/T2 and T1 sequences.

Modic 1 lesions have the potential to mimic infectious changes. The distinction between a Modic 1 lesion and a spondylodiscitis can be made by the lack of abnormal disc signal or disc hypointensity on T2-weighted images. End plates may show enhancement in both degenerative and infectious alterations. Topographically, Modic 1 lesions are usually found at the distal lumbar tract. In population studies, the prevalence of Modic changes is 13% at lumbar level, and the Modic 2 lesions are largely prevalent (82%) over Modic 1 (10.8%) and Modic 3 (7.2%) [24].

Modic lesions are easily detected at cervical level and were are associated with neck pain and disc degeneration from 5 to 40% of the cases. Also at this level, Modic 2 changes are the most frequent [25].

The aetiology of Modic type 1 lesions is still debated. Some authors have hypothesized that these lesions could be secondary to anaerobic germ infection [26]. There is certainly an inflammatory response of the annulus fibrosus characterized by neovascularization around the extruded nucleus pulposus following herniation [27]. The intervertebral degeneration of the disc is characterized by a gradual dehydration of the disc itself and appears in MRI as loss of the normal hyperintensity of the nucleus pulposus in T2 sequences with consequent loss of disc height. A severely degenerated disc may, however, reveal a T2 signal hyperintensity. In these cases, a degenerated disc may be difficult to differentiate from infectious spondylodiscitis. Degenerative endplate degeneration in Modic 1 lesions may be similar to the BME of endplates observed in spondylodiscitis [28]. Similar to spondylodiscitis, inflammatory neovascularization within the subchondral bone is believed to be the cause of BME in Modic 1 lesions [29].

### Acute symptomatic calcific discitis

Calcific discitis (CD) is a well-recognized entity mainly in paediatric patients, but recently it has also been reported in the adult population, present in 5% of chest x-rays and 6% of abdominal x-rays [30]. CD is generally an asymptomatic condition, associated with underlying predisposing diseases such as hyperparathyroidism, chondrocalcinosis, or hemochromatosis [31]. CD most commonly occurs in the cervical segment of the spine [32], followed by the thoracic segment, rarely present in the lumbar segment [33]. The diagnosis of CD is based on imaging techniques. CD in MRI classically shows a low signal central focal lesion in the disc on standard T1- and T2-weighted spin-echo sequences [34]. The MRI may also reveal a swollen intervertebral disc. Early CD may not be radiographically evident and an edematous swollen MRI disc may be the only indication of a CD [35]. There may be diffuse reactive edema of the adjacent vertebra. Contrast enhancement during CD may be seen within the vertebral body if the calcific herniated disc in the vertebral body itself [36].

### Spinal gout

The deposition of monosodium urate in the spine is considered a rare manifestation of gout [37]; however, axial disease seems overlooked since alterations suggestive of monosodium urate deposition in axial structures are detectable in the 17% of gouty patients [38]. Any segment of the spine may be involved in its components (vertebral bodies, pedicles, lamina, ligaments, interapophyseal cartilage, epidural and intradural spaces). The lumbar region is the most common involved spinal site (78% of the spinal locations) [38]. The clinical symptoms of spinal gout are non-specific and very varied, ranging from back pain to neurological deficits. Gout involving the endplates of two contiguous vertebral bodies and the intervertebral disc is a condition that can mimic spondylodiscitis [39]. The diagnosis of tophaceous spinal gout is extremely difficult, and the most accurate test to confirm its diagnosis is histological examination. Computed tomography (CT) can help to visualize changes in bone and soft tissue caused by tophi, which appear as a low-density area [40].

Dual energy computed tomography (DECT) can provide additional information respect to conventional CT in the diagnosis of gout in spinal structures, with a sensitivity between 78 and 100% and a specificity between 89–100% in detecting monosodium urate deposition [41].

MRI appears to be sensitive but not specific for the diagnosis of spinal gout, but has the advantage of representing soft tissue changes [42]. Spinal tophi appear at MRI as homogeneous areas of medium to low signal intensity on T1-weighted images. On T2-weighted images, the signal intensity of the tophi varies from homogeneous hyperintensity to homogeneous hypointensity. The signal intensity variations of tophi depend mainly on their degree of hydration and the relative homogeneity of the magnetic field within them. These may be due to the presence of calcifications, mature fibrous tissue or hemosiderin deposits within the tophi [43]. After administration of contrast medium, the tophi show a homogeneous or heterogeneous marginal enhancement. The enhancement of tophi depends on the amount of vascularized inflammatory fibrous tissue. The main differences between spinal gout and spondylodiscitis are the presence, in gout, of spondylolisthesis due to bone erosion of the pars interarticularis and the facet joint, smooth bone erosion predominantly localized in the L5 lower end plate rather than irregular changes, and normal intensity destruction of the bone marrow signal of adjacent vertebrae*.*

### Destructive spondyloarthropathy in long-term haemodialysis

Destructive spondyloarthropathy (DSA) is increasingly reported as a serious complication of long-term haemodialysis, affecting 8–18% of dialysis patients [44]. DSA affects single or multiple, contiguous or distant spinal levels. Atlantoaxial involvement is not common [32]. DSA is a radiographic diagnosis based on three findings characterized by severe narrowing of the intervertebral disc spaces, erosions and cystic changes of adjacent vertebral plates, and the absence of significant osteophytosis. The MRI is useful to exclude the presence of infection (absence of high signal strength in T2-weighted sequences) [45]. In MRI, DSA shows alterations generally characterized by low signal intensity in the affected spinal segments in T2-weighted images, even if T2 images with abnormal high signal intensity have been described [46].

## Infectious spondylitis

Spondylodiscitis is generally infection sustained by a single germ, and Staphylococcus aureus is the predominant aetiological agent, affecting about half of non-tubercular infections [47]. The causative agents of granulomatous infections are Mycobacterium tuberculosis and Brucella. Fungal or parasitic infections are rare. The spectrum of clinical manifestations is broad, but pain is the main symptom, regardless of the aetiological agent. Men are affected about twice as often as women, diabetes mellitus is a risk factor.

### Pyogenic spondylodiscitis

**Fig. 6:**
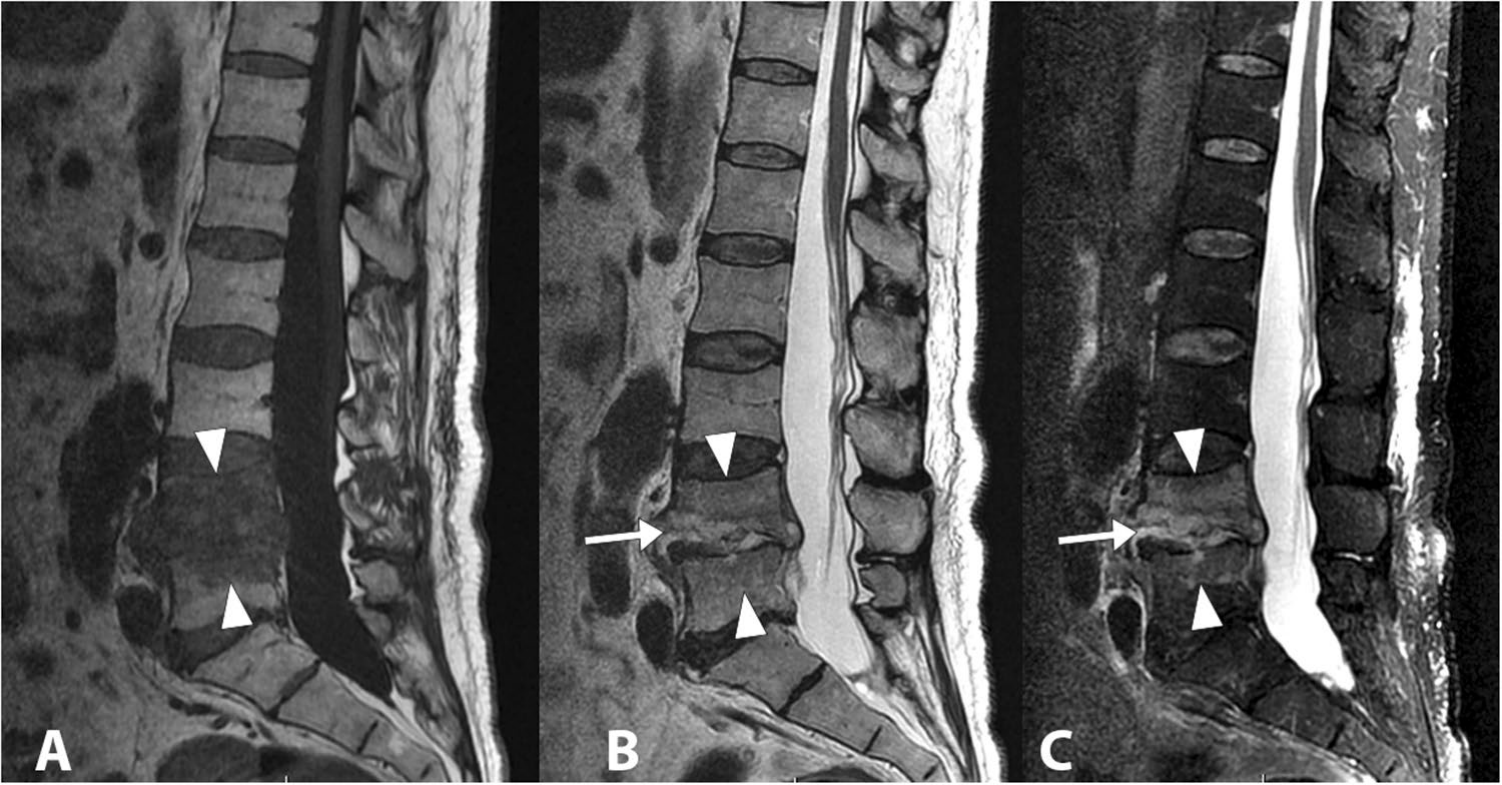
Lumbar (L4-L5) spondylodiscitis caused by Staphylococcus aureus. The whole vertebral body of L4 and the upper portion of L5-vertebral body show an altered signal intensity in the T1-weighted image (**a**), in the T2-weighted image (**b**) and in the fat saturated T2-weighted image (**c**) (arrowheads). The L4–L5 disc is involved and it appears thinned, with an increased signal intensity in the T2-weighted and fat saturated T2-weighted images (arrows)

**Fig. 7:**
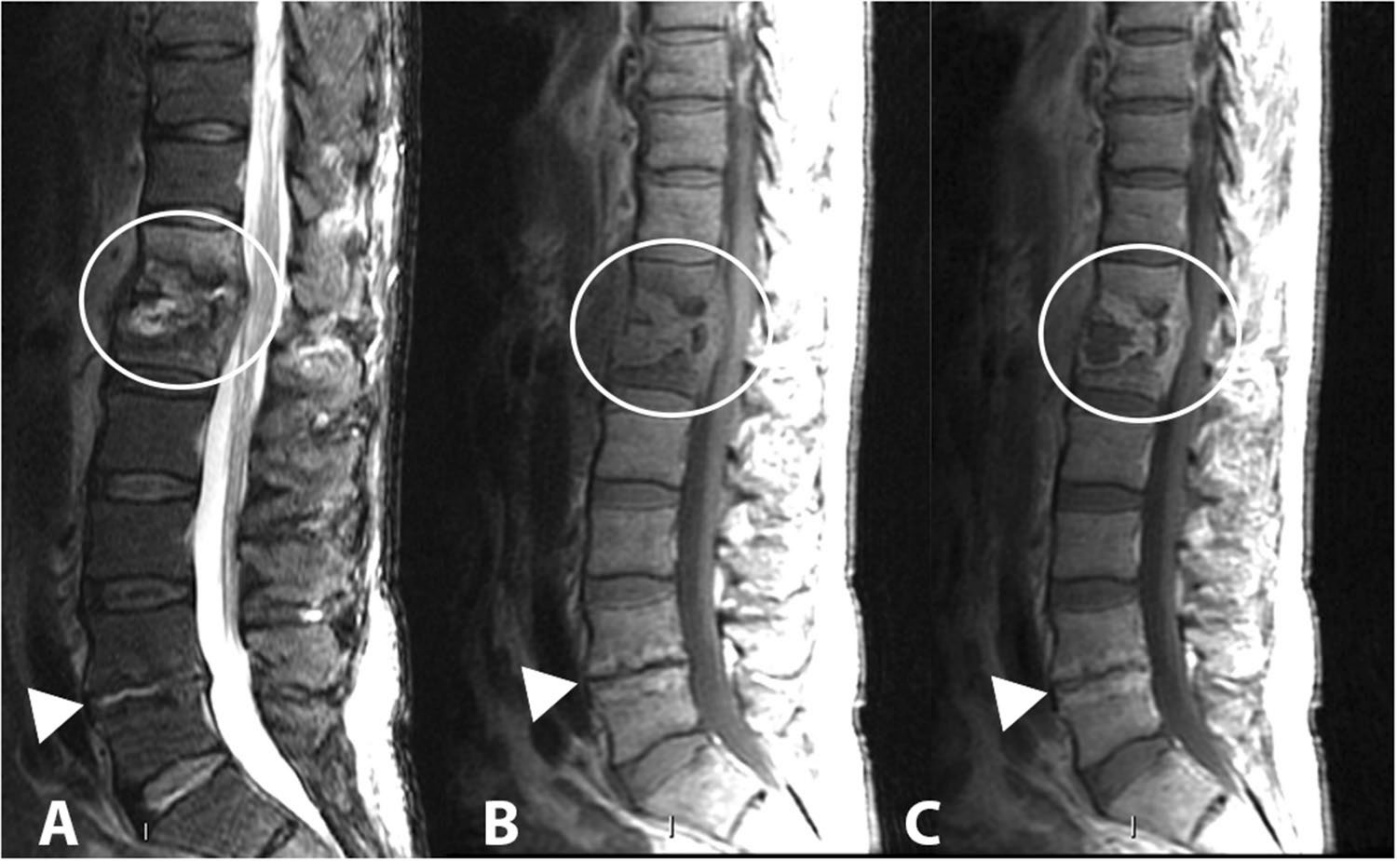
Spondylodiscitis caused by Staphylococcus aureus. The D12-L1 disc and the adjacent vertebral bodies appear markedly involved in the infectious process. The fat saturated T2-weighted sequence (**a**) and the sagittal T1-weighted sequence (**b**) show an altered signal intensity of the affected segments. The sagittal T1-weighted image after gadolinium administration (**c**) highlights a significant contrast enhancement of the affected area, with a posterior extension into the epidural space (white circle). In addition, a Modic 2 lesion can be observed at L4/L5, with hypointense signal in STIR image (**a**) and high signal intensity in T1-weighted images (**b, c**) (arrowhead)

Pyogenic spondylodiscitis generally occurs by haematogenous spread from distant infectious foci in the body, more rarely by contiguity (e.g. oropharynx, pleural space, abdominal cavity) [48]. Most cases are supported by gram-positive cocci such as Staphylococcus aureus (Figs. 6, 7), followed by Staphylococcus epidermidis, and Streptococcus spp. [49]. Among the gram-negative bacteria, Escherichia coli is the most commonly isolated pathogen, followed by Enterobacter cloacae, Haemophilus influenzae, Klebsiella pneumonia, and Salmonella enterica. Patients with gram-negative haematogenous spondylodiscitis, compared to those with gram-positive infections, tend to be older individuals with a history of cancer and a recent positive anamnesis for symptomatic urinary tract infection. Gram-negative forms also show a lesser tendency to the formation of abscess cavities [50].

**Fig. 8:**
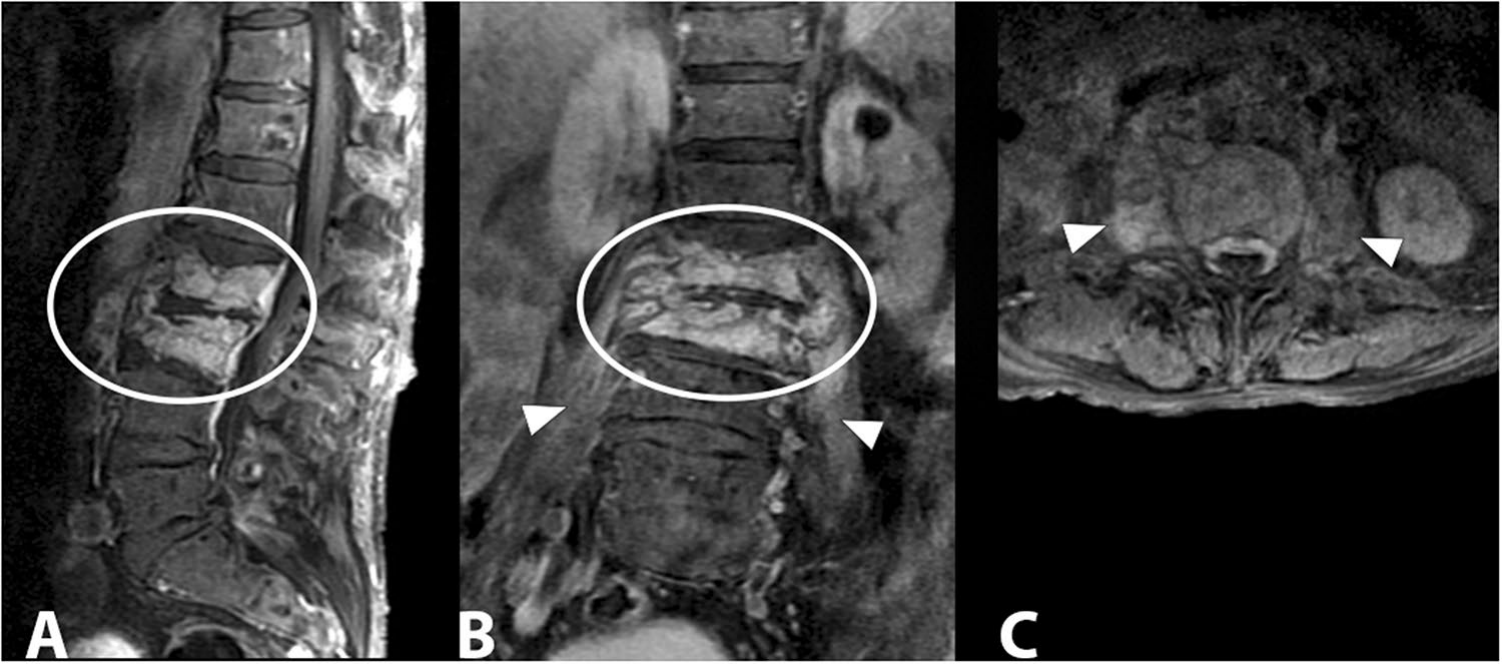
Gram-positive (Streptococcus spp) lumbar spondylodiscitis. Sagittal (**a**), coronal (**b**) and axial T1 fat suppression (**c**) images after administration of contrast media show enhancement of the L2 and L3 vertebrae and paravertebral soft tissues (white oval) with involvement of the psoas muscles bilaterally (arrowheads)

**Fig. 9:**
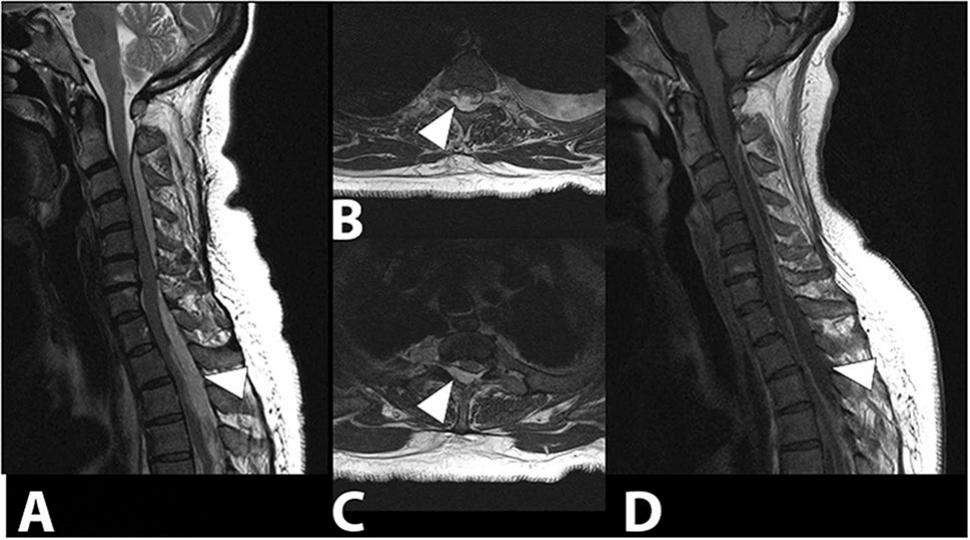
Sagittal (**a**) and axial T2-weighted images (**b**, **c**) and sagittal T1-weighted fat suppression image with contrast (**d**). Staphylococcus aureus posterior cervico-dorsal epidural abscesses that compress and displace the spinal cord anteriorly can be observed (arrowhead)

**Fig. 10:**
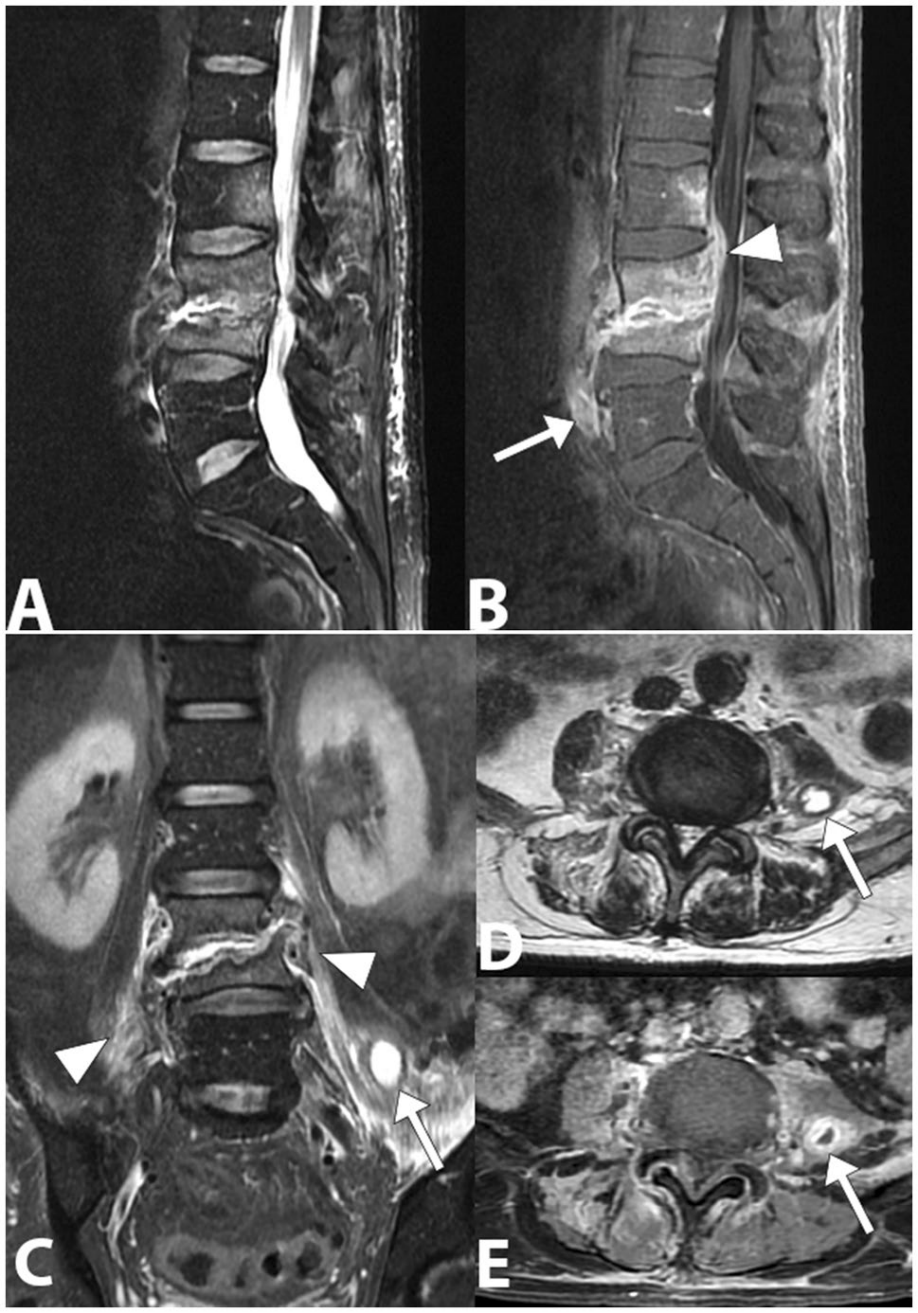
Gram-negative (Escherichia coli) spondylodiscitis. Sagittal lumbar spine MRI image (**a**) showing a  high bone marrow signal and partial collapse of the vertebral bodies L3 and L4, destruction of the L3–L4 disc and loss of the definition of the end plate on both sides of the disc. In the T1-weighted sagittal image obtained with saturation of the fat signal and after the injection of the contrast medium (**b**), a better definition of the involvement of the paravertebral (arrow) and epidural space with narrowing of the vertebral canal (arrowhead) can be observed. The posterior L2 vertebral body is also involved. In coronal STIR image (**c**), axial T2- weighted (**d**) and axial T1- weighted with fat saturation and gadolinium (**e**), edema and inflammatory exudate can be observed in the paravertebral soft tissue (arrowheads) and abscesses in the left psoas muscle (arrow)

The clinical presentation of spondylodiscitis is variable, but the cardinal clinical sign is the presence of severe pain associated with muscle contracture (present in more than 90% of cases). Fever and neutrophil leukocytosis are present in 40%–50% of cases. Erythrocyte sedimentation rate (ESR) and C-reactive protein (CRP) are almost always increased [51]. Lumbar segments are more frequently involved (48%) (Fig. 8), followed by dorsal (35%), and cervical (6.5%) segments (Fig. 9) [52]. Cervical localization is a rare, but often site of spinal cord compression complications [49]. Possible abscess complications concern the lumbar spine in 2/3 of the cases, with prevalently paravertebral localization (Fig. 10), or the thoracic region in 1/3 of the cases, with prevalently epidural localization.

MRI is the most sensitive technique in the diagnosis of spondylodiscitis. Although it is the most sensitive tool for early detection of signs of infection, MRI results may lag behind clinical symptoms. If the clinical picture is uncertain, an MRI one week after the previous one may be useful to show an evolution. Sagittal-weighted T1 images typically show hypointense and poorly defined vertebral bone marrow in contiguous vertebral bodies. Intervertebral disc space is involved with loss of end plate definition on both sides of the disc. Weighted spin-echo T2 images detect increased water content expression of inflammatory exudate. Intravenous gadolinium contrast is usually administered in all suspected cases of spondylodiscitis. In patients who cannot receive gadolinium contrast, diffusion-weighted imaging (DWI) can be used.

### Infectious granulomatous diseases

Some infectious processes within the spinal elements can lead to the formation of granulomas [53]. The organisms causing granulomatous inflammation include various bacteria, fungi or other parasites. Among the bacteria, those most frequently found are Mycobacterium tuberculosis and Brucella. The onset of granulomatous infections is often insidious and often leads to a late diagnosis.

#### Tubercular spondylodiscitis

Spinal involvement in tuberculosis (Pott's disease) occurs mainly by haematological spread. The clinical presentation of vertebral tuberculosis is insidious, with symptoms that can last up to 3 years before diagnosis [54]. From an epidemiological point of view, tubercular spondylodiscitis is significantly more common in patients under 40 years of age than in patients older [55].

Thoracolumbar region is the most commonly affected site, while the cervical and sacrum regions are less commonly involved. Usually more than one vertebra is affected because of its segmental arterial distribution and subligamentous spread of the disease.

**Fig. 11:**
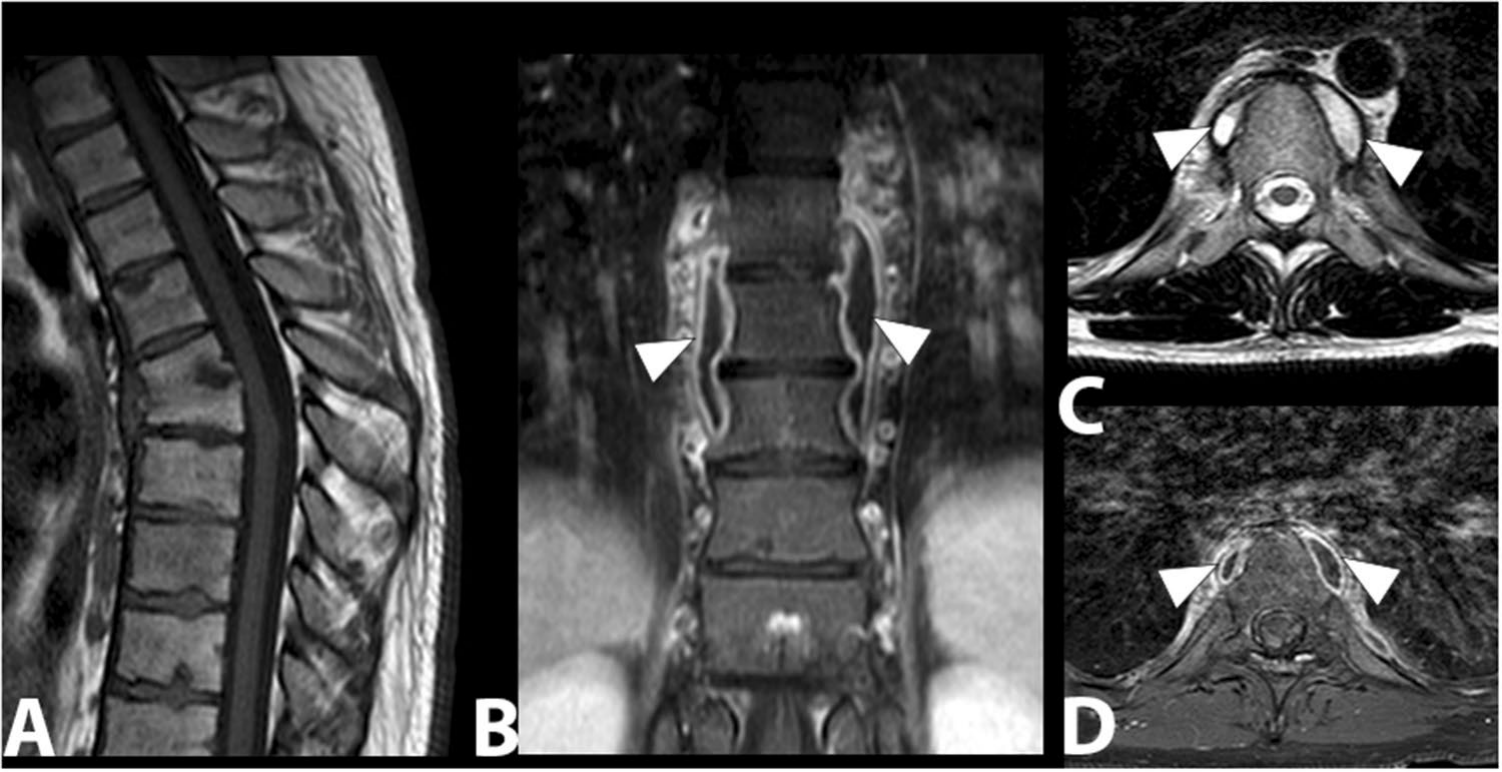
D6–D7 tubercular spondylodiscitis. In the sagittal T1 image (**a**)**,** morphostructural alterations of the D6 and D7 with collapse and wedging of the vertebral bodies, accentuation of the physiological dorsal kyphosis, absence of the disc space and partial vertebral fusion can be observed. In the coronal T1 image with contrast (**b**), T2 (**c**) and T1 axial with contrast (**d**), bilateral paravertebral infectious collections are detected (arrowheads)

**Fig. 12:**
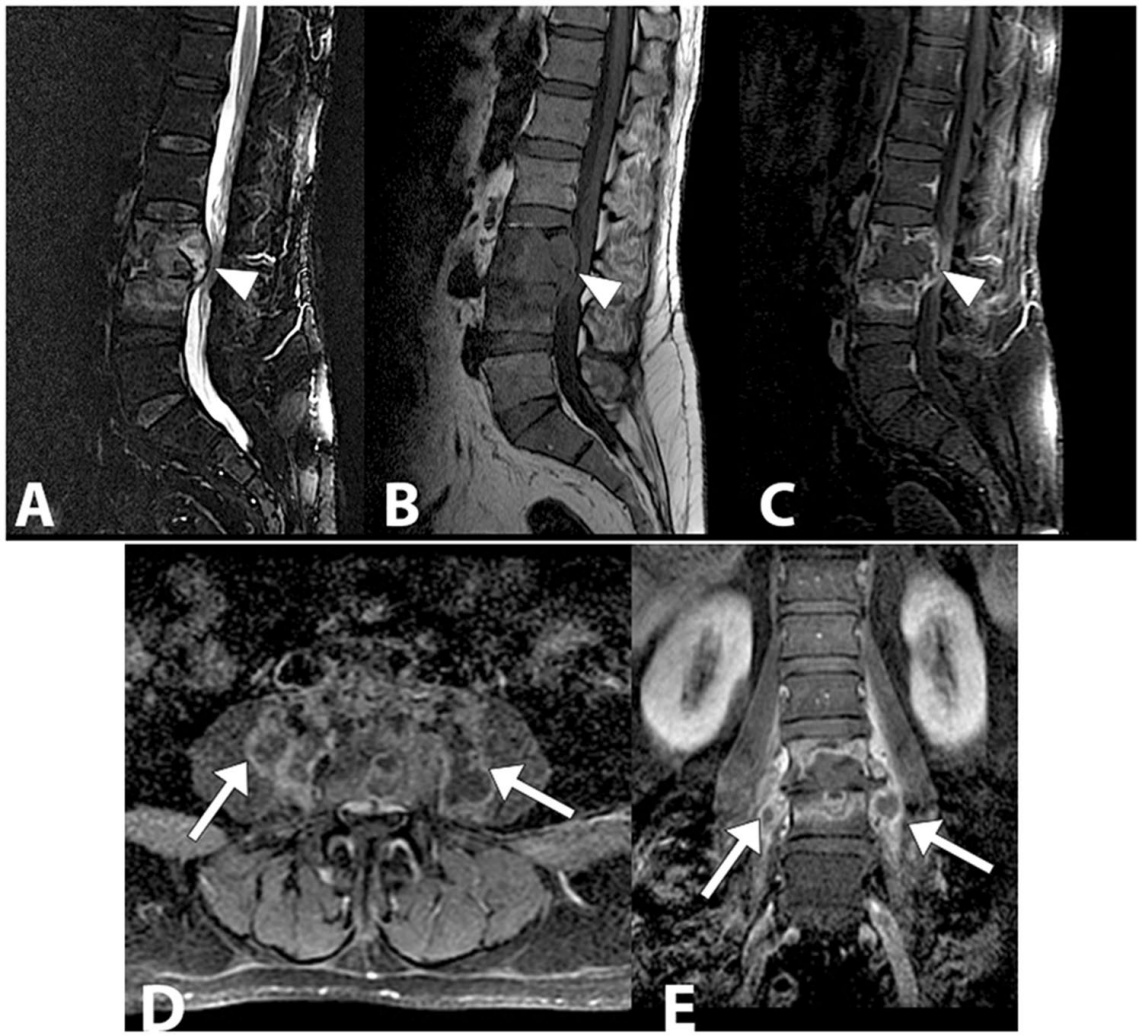
Lumbar tubercular spondylodiscitis. Sagittal STIR (**a**), sagittal T1 without contrast (**b**), sagittal T1 with contrast (**c**) images demonstrating severe osteo-structural alteration of the L3 vertebral body with invasion of the epidural space, dural sac and spinal roots compression (arrowhead). Axial (**d**) and coronal (**e**) T1-weighted images with contrast show paravertebral abscesses affecting the psoas muscles bilaterally (arrows)

**Fig. 13:**
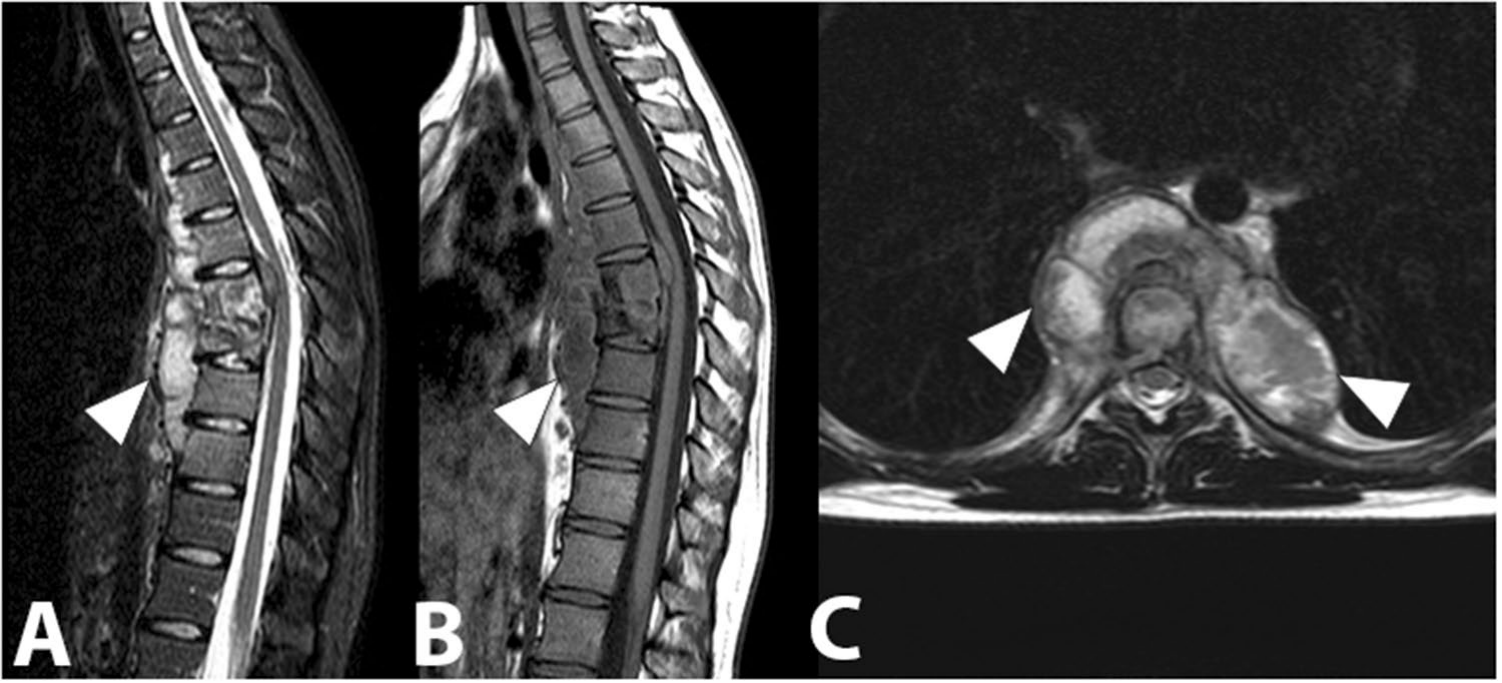
Tubercular spondylodiscitis with altered signal and morphology of vertebral bodies D7 and D8 in sagittal STIR (**a**) and T1-weighted images (**b**), absence of intervertebral disc space D7–D8 and presence of paraspinal abscesses extended from D3 to D9, better assessable in axial images (**c**) (arrowheads)

Tubercular spondylodiscitis usually begins in the antero-inferior part of the vertebral body. The spread of infection occurs under the anterior longitudinal ligament, a structure that involves adjacent vertebral bodies. The narrowing of the disc space occurs secondarily and is not as pronounced as in pyogenic infections. The relative saving of the intervertebral disc appears to be due to the lack of proteolytic enzymes in Mycobacterium tuberculosis [56]. MRI is the diagnostic technique of choice, more sensitive than X-ray and more specific than CT in the diagnosis of spinal tuberculosis. MRI demonstrates involvement of vertebral bodies, disc destruction, cold abscess, vertebral collapse and spinal deformities (Fig. 11). Compared to pyogenic infections, the disc may not show a signal increase in T2-weighted images [57]. The involvement of posterior elements is more common in tubercular infections than in pyogenic infections. Posterior lesions enter into differential diagnosis with neoplastic lesions, particularly when there is relative preservation of disc space. Tubercular infections classically spread to adjacent ligaments and soft tissue in an antero-lateral direction (Fig. 12). The paravertebral abscesses are surrounded by a rim characterized by a robust and irregular enhancement, which can be seen in MRI. These abscesses tend to be larger in tubercular infections than in pyogenic infections (Fig. 13).

Abscess formation is common and can grow to a very large size. The site of cold abscess depends on the region of the vertebral column affected. In the cervical region, the pus accumulates behind prevertebral fascia to form a retropharyngeal abscess. The abscess may track down to the mediastinum to enter into the trachea, oesophagus, or the pleural cavity. In the thoracic spine, the cold abscess usually presents as a fusiform or bulbous paravertebral swellings. At lumbar vertebrae, cold abscesses most commonly present as a swelling in the groin and thigh and pus collection can spread to the gluteal region [58, 59].

### Brucellar spondylodiscitis

Brucellosis can account for up to about half of spinal infections in areas where the zoonosis is endemic, being the predominant cause in some case series in the Mediterranean basin and the Middle East [55]. The aetiological agent is Brucella melitensis, an intracellular bacterium [60]. Osteoarticular involvement is a common complication of brucellosis, found in up to 85% of patients [61]. In decreasing order of frequency, spinal involvement concerns the lumbar (60%), sacral (19%) and cervical (12%) vertebrae [62]. Spondylodiscitis during brucellosis can be multifocal. This type of involvement can be observed in 3–14% of patients [63]. Spinal brucellosis usually starts from the upper terminal vertebral end plate, but sometimes the lower terminal end plate may also be involved. In MRI, the lesion is detectable as a destructive aspect at the antero-superior vertebral angle accompanied by prominent osteosclerosis, and it is a pathognomonic sign (Pedro Pons' sign) [64]. The intervertebral disc can be infected without spondylitis, being only a discitis. Epidural abscess is a rare complication of spinal brucellosis, but can lead to severe neurological outcomes.

### Fungal vertebral infections

Fungal spondylodiscitis is rare (up to 1.6%) even in larger case series; however, the incidence of these conditions is increasing as the population of immunocompromised patients increases [65]. Candida spp., Aspergillus spp. and Cryptococcus neoformans are present worldwide, while dimorphic fungi such as Coccidioides immitis and Blastomyces dermatitidis are only endemic in some geographical areas. Spinal fungal infection includes spondylodiscitis, osteomyelitis and meningitis.

MRI features, such as focal partial soft tissue abnormality and partial involvement of the disc/endplate, in combination with clinical features may help to predict fungal discitis/osteomyelitis [66].

#### Candida

**Fig. 14:**
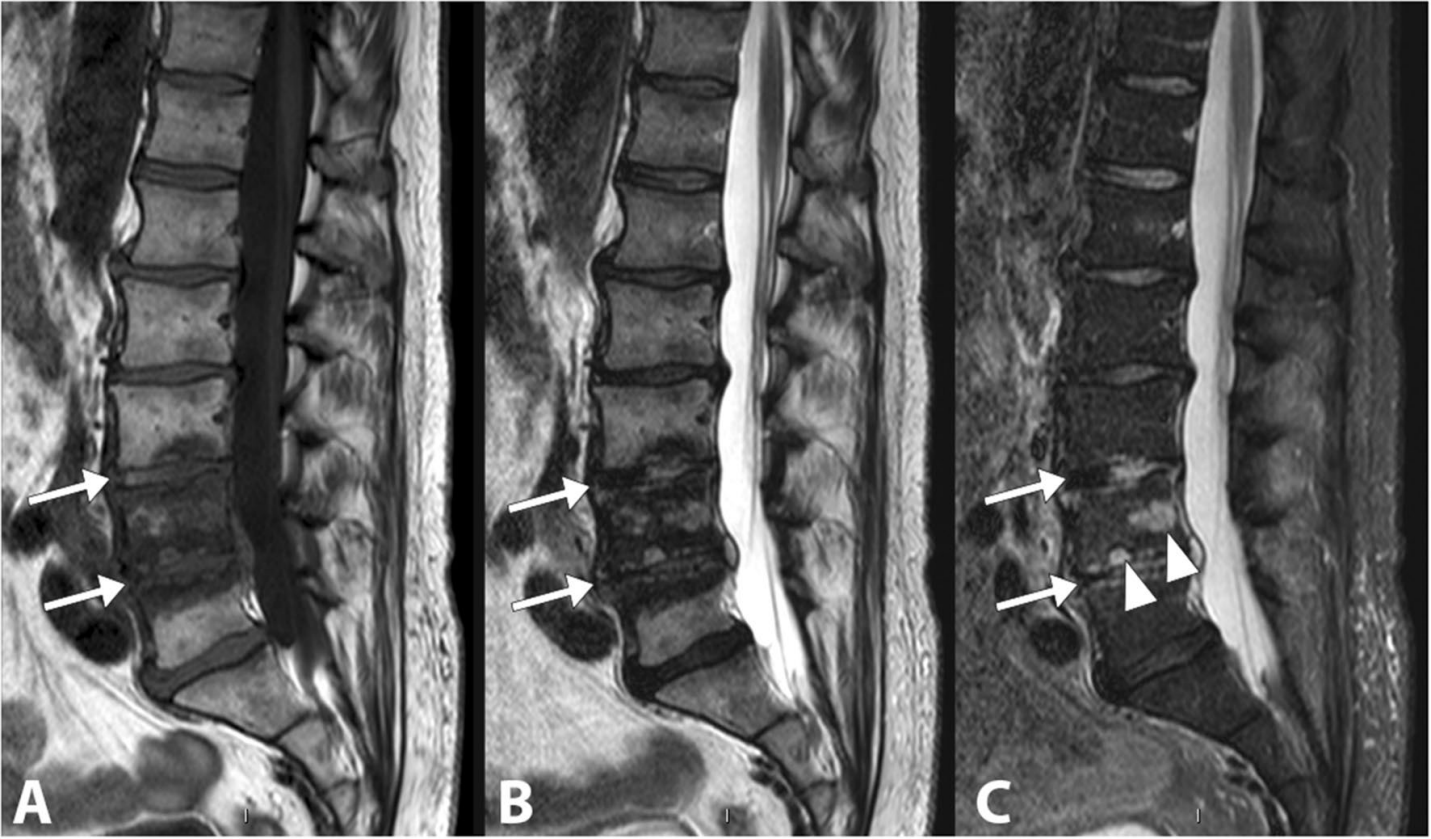
Gradually resolving lumbar (L3–L4–L5) spondylodiscitis caused by Candida  tropicalis. The sagittal T1-weighted (**a**), T2-weighted (**b**) and fat saturated T2-weighted (**c**) images show an altered signal intensity in correspondence with the L3–L4 and L4–L5 discs (arrows). Some infectious foci are still visible in the vertebral body of L4 (arrowheads)

Although there are at least 10 species of Candida pathogenic to humans, Candida albicans is responsible for more than half of all cases of spondylodiscitis supported by Candida spp., followed by Candida tropicalis (19%) (Fig. 14), and Candida glabrata (formerly Torulopsis glabrata, 14%). Spondylodiscitis supported by Candida glabrata is becoming increasingly common. Overall, Candida spp. is responsible for 0.7–2.7% of spinal infections [67]. The lower dorsal and lumbar vertebral segments are the most frequently involved sites. The few reported cases occurring at a higher spinal or sacral level are anecdotal. At diagnosis, 83% of patients have been complaining of back pain for at least one month, while only 32% of patients are febrile [68].

MRI is the imaging mode of choice and the vertebral bodies and discs typically present hypointense in T1 and hyperintense in T2. Administration of contrast agent can improve sensitivity and specificity particularly in early infections [69].

#### Aspergillus

Aspergillus fumigatus is the most frequently isolated species in bone infections. The lumbar region is the main area (63%) of bone involvement, followed by extra-axial regions such as tibia, ribs, wrist, sternum, pelvis and knee [70]. Spondylodiscitis supported by Aspergillus shares several common features with other causes of pyogenic vertebral spondylodiscitis, including male preference, predominance of lumbar involvement, and pain as a symptom of onset.

In the presence of an Aspergillus infection, discs lack signal hyperintensity on T2-weighted and STIR images, due to the presence of the paramagnetic and ferromagnetic elements within the fungi and the nuclear cleft may be preserved, a very uncommon finding in pyogenic spondylitis.

As in tuberculosis, subligamentous spread of abscess and multilevel involvement of the spine can be observed and the intervertebral disc may be spared from invasion and inflammatory changes [71].

#### Cryptococcus

Cryptococcus neoformans is the fungus that most commonly causes central nervous system diseases in humans. Spinal involvement occurs in up to 10% of adult cryptococcosis patients, with characteristics similar to those of cold abscesses of tubercular spondylodiscitis [72]. Vertebrae are the most common site of bone involvement in cryptococcosis, the lumbar region being the most affected segment followed by the cervical region [73].

#### Coccidioidomycosis

Coccidioides immitis causes bone localization in up to 50% of patients with diffuse disease [74]. The most involved articular segments are the spine, ribs and pelvis [75]. Vertebral localizations may involve one or more vertebral bodies, paraspinal tissue and contiguous ribs. Intervertebral discs are relatively spared. Vertebral collapse and fistulae are uncommon late manifestations. In contrast to tuberculosis, in coccidioidomycosis spondylitis the gibbous deformity is not common, although reported [76]. MRI images are not specific, and diagnosis is usually made by biopsy in patients from endemic areas.

#### Blastomycosis

Skeletal localizations are observed in 14–60% of cases of diffuse blastomycosis. The spine is the most commonly involved skeletal site, followed by the skull, ribs, tibia and bones of the foot and wrist [77]. The lower dorsal spine and lumbar segments are the most affected regions, similarly to tuberculosis. The anterior aspect of the vertebral body is usually involved in the early stages. Even non-adjacent vertebrae can be affected by infection along the anterior longitudinal ligament. This can cause gross deformities of the vertebral spine. Paravertebral abscesses or iliopsoas muscle can be documented.

### Parasitic spinal infections

Several parasites affecting the central nervous system can affect the spine. The most common of these diseases is cysticercosis. In endemic regions, schistosomiasis is a common cause of spinal involvement. Toxoplasmosis is a pathogen frequently found in immunodepressed patients. Echinococcosis and hydatidosis are also emerging parasitic diseases in some parts of the world. Although the definitive diagnosis of a spinal parasitosis is usually confirmed by histological biopsy examination, the clinical suspicion is generally based on a combination of epidemiological, clinical, serological, and neuroimaging features.

#### Cysticercosis

Cysticercosis, the most common central nervous system parasitosis, is caused by Taenia solium [78]. Neurocysticercosis involving the spinal cord is extremely rare. Because of the mass effect and limited space within the canal compared to intracranial space, spinal cysticercosis is more likely to cause neurological impairment. Neurological deficits are secondary to the mass effect of cysts and the inflammatory reaction after treatment.

#### Schistosomiasis

Schistosomiasis is a parasitosis caused by Schistosoma platyhelminthes. Schistosoma japonicum, Schistosoma mansoni and Schistosoma haematobium can cause pathology in humans [79]. Spinal cord injuries are often caused by Schistosoma mansoni and Schistosoma haematobium. MRI is the imaging mode of choice to assist in the diagnosis of schistosomiasis with spinal localization. MRI can document an enlargement of the spinal cord caused by the formation of intramedullary granulomas, particularly in the lower spinal cord region and the conus medullaris region [80]. Schistosomal intramedullary granulomas are usually seen isointense in relation to the cord, or as heterogeneous hyperintense lesion with an unclear boundary, or as multiple patchy nodular lesions resembling a string of beads mainly in the ventral spinal cord which may be significantly enhanced. Atrophy of the spinal cord may be seen in longstanding cases [81].

#### Toxoplasmosis

Toxoplasmosis is the most common opportunistic infection of the central nervous system [82]. The disease is caused by Toxoplasma gondii, obligate intracellular protozoon. At MRI, the lesions are hyperintense on T2-weighted sequences and show increased contrast after gadolinium administration on T1-weighted sequences. Localized intramedullary ring-enhancing lesions are commonly seen in MRI during toxoplasmosis [83]. In case of a regular spinal cord size, the presence of an abnormal spinal signal may suggest a vacuolar myelopathy. An enlargement of the spinal cord is suggestive of the presence of Toxoplasma myelitis.

#### Echinococcosis/hydatidosis

Echinococcus granulosus is a parasitic zoonosis commonly involving liver and lungs. The skeletal localization of hydatid cysts is a rare occurrence, when it occurs it affects the spine in almost 50% of cases [84]. Spinal hydatid cysts account for 1% of all cases of hydatid disease [85]. The most common spinal site of extramedullary intradural hydatid cysts is the dorsal region (46.5%), followed by the lumbar (30.2%) and cervical region (9.3%). Hydatid cysts can be unilocular or multilocular, the first type being more common (57.5%) [86]. In hydatid disease, spinal involvement is classified into five groups: (1) primary intramedullary hydatid cyst; (2) intramedullary extramedullary intradural hydatid cyst; (3) extradural intraspinal hydatid cyst; (4) vertebral hydatid disease; (5) paravertebral hydatid disease. The first three types of this group are considered rare [87].

On MRI T1-weighted images, the hydatid cyst wall may be isointense or give a slightly lower signal than its content, and T2-weighted images show a low-intensity border surrounding the homogeneous high signal cyst content. The hydatid cyst wall shows a slight contrast enhancement. The low signal rim on T2-weighted images results from reactive fibrosis and degeneration around the parasite membrane. The T2-weighted images give information on the vitality of the hydatid cyst: a decrease in the high signal and an increase in the low signal of the collapsed cystic walls indicate a failing cyst.

## Differential diagnosis

MRI allows to identify some specific signs of the various infectious spondylodiscitis. The main features of the pyogenic spondylodiscitis are the involvement of the lumbar spine, poor enhancement of the paravertebral tissues, diffuse/homogeneous vertebral contrast enhancement, low degree destruction of the vertebral bodies, hyperintense/homogeneous signal of the vertebral bodies on T2-weighted images.

Tubercular spondylodiscitis is mainly characterized by relative disc preservation and other features such as intraosseous abscesses, large paravertebral abscesses, skip lesions, contiguous subligamentous diffusion, involvement of posterior elements and invasion of the vertebral canal and nerve roots [88] (Table 2). In tubercular spondylodiscitis, the size of paraspinal abscesses is usually larger than that of brucellar forms. The involvement of posterior spinal elements, in particular the involvement of pedicles, is generally not a feature of spinal tuberculosis [89, 90]. The upper lumbar spine and lower thoracic spine are the most frequently involved sites. More than one vertebra is typically affected, and the vertebral bodies are more frequently affected than the posterior arches.

**Table 2:** Clinical and MRI features to differentiate tubercular from pyogenic infections ( modified from*:* Frel et al. [95])

	Pyogenic spondylitis	Tuberculous spondylitis
*Patient characteristics and clinical symptoms*		
Age	Relatively old	Relatively young
Duration to diagnosis	Relatively short symptom to diagnosis interval	Relatively long symptom to diagnosis interval
History	Recent distant bacterial infection or previous spinal surgery	History of TBC infection or current extraspinal manifestations
Onset	Acute or subacute	Subacute
Fever	More frequent associated high fever, acute sepsis	Intermitted fever
ESR, CRP, WCC	Markedly increased	Mild increased
*MRI features*		
Involvement of vertebral bodies	Involvement ≤ 2 vertebral bodies	Multiple body involvement
Severity of destruction of vertebral bodies	Infrequent and mild to moderate	Frequent and more severe
Disc destruction	Severe to complete disc destruction	Normal to mild disc destruction
Loss of cortical definition	Absent	Present
Areas of paraspinal enhancement	Poorly demarcated contrast	Well-demarcated contrast
Vertebral signal in T2 images	Hyperintense/homogeneous	Heterogeneous
Vertebral enhancement	Diffuse/homogeneous	Focal/heterogeneous
Paraspinal abscess	39–40% of cases	75% of cases
Epidural abscess	11–15% of cases	56–60% of cases
Abscess wall	Thick and irregular	Thin and smooth
Meningeal enhancement at the affected vertebral level	28–30% of cases	> 75% of cases
Subligamentous spread to 3 or more vertebral bodies	Absent	Present
Spinal deformity	Absent	Present
Thoracic spine involvement	Absent	Present

*MRI* magnetic resonance imaging, *ESR* erythrocyte sedimentation rate, *CRP* C-reactive protein, *WCC* white cell count

An almost intact vertebral architecture is observed in brucellar spondylodiscitis, despite evidence of widespread vertebral infection. An increase in the signal intensity of the disc on T2-weighted and high-contrast images, as well as the involvement of the articular facets, is also typical [91]. Paravertebral abscesses tend to be smaller than those of tubercular infections [92]. In the acute (< 3 months) and subacute (3–12 months) phases, brucellar spondylodiscitis may mimic tuberculosis. In these phases, the more homogeneous intensity of the high-intensity vertebral signal on STIR sequences and the almost intact vertebral height seem to suggest the diagnosis of brucellar spondylodiscitis instead of tuberculosis [93].

Most fungal spinal infections have no MRI imaging peculiarities. The destruction of the vertebral body in this type of disease may mimic tubercular spondylitis. The absence of signal enhancement on T2-weighted images and low or undetectable enhancement after contrast administration may be MRI features of fungal infections [94]. MRI may overestimate the extent of infected tissue. Therefore, additional information from conventional radiology or CT may be required to define the actual amount of tissue necrosis. Table 2 summarizes the characteristics that differentiate individual spondylodiscitis types [95].

## Conclusions

Spondylodiscitis involve the vertebral bodies, the intervertebral disc, and may involve the paravertebral structures and the spinal canal. If not recognized and treated early, morbidity and mortality rates are potentially high. The incidence of spondylodiscitis has increased in recent years due to an increase in the immunodepressed population but also due to improved diagnostic accuracy. However, the diagnosis remains challenging because the disease can have an insidious onset, with sometimes non-specific clinical features. MRI is the most sensitive technique for the diagnosis of spondylodiscitis. It allows diagnosis in the absence of radiographic signs and can provide indications on the aetiological agent. However, specific differentiation of spondylodiscitis subtypes based on MRI finding seems to be difficult, particularly when some of the classic imaging features are absent or when there are unusual patterns of infectious spondylitis. Moreover, non-infectious inflammatory diseases and degenerative disease may simulate spinal infection.

According to the role of MRI in the follow-up of treated patients with spondylodiscitis, the 2015 American Society guidelines do not recommend follow-up MRI in patients in whom a favourable clinical and laboratory response to antimicrobial therapy was observed [96].

The limits of MRI are mainly the limited availability and accessibility of the machines, and the execution time of examinations. Future research should focus on further validation of lesions detectable in MRI in extensive prospective studies.

## Data Availability

Not applicable.
